# The Hemagglutinin-Esterase Fusion Glycoprotein Is a Primary Determinant of the Exceptional Thermal and Acid Stability of Influenza D Virus

**DOI:** 10.1128/mSphere.00254-17

**Published:** 2017-08-09

**Authors:** Jieshi Yu, Busha Hika, Runxia Liu, Zizhang Sheng, Ben M. Hause, Feng Li, Dan Wang

**Affiliations:** a Department of Biology and Microbiology, South Dakota State University, Brookings, South Dakota, USA; b Department of Biochemistry and Molecular Biophysics, Columbia University, New York, New York, USA; c Department of Systems Biology, Columbia University, New York, New York, USA; d Cambridge Technologies, Inc., Worthington, Minnesota, USA; e Department of Veterinary and Biomedical Sciences, South Dakota State University, Brookings, South Dakota, USA; f BioSNTR, Brookings, South Dakota, USA; Emory University School of Medicine

**Keywords:** hemagglutinin-esterase fusion protein, acid stability, influenza virus, thermal stability

## Abstract

Influenza D virus (IDV) is unique among four types of influenza viruses in that it utilizes cattle as a primary reservoir. The thermal and acid stability of IDV were examined and directly compared with those of influenza A virus (IAV), influenza B virus (IBV), and influenza C virus (ICV). The results of our experiments demonstrated that only IDV had a high residual infectivity (~2.5 log units of 50% tissue culture infective dose [TCID_50_]/ml) after a 60-min exposure to 53°C in solution at a neutral pH, and remarkably, IDV retained this infectivity even after exposure to 53°C for 120 min. Furthermore, the data showed that IDV was extremely resistant to inactivation by low pH. After being treated at pH 3.0 for 30 min, IDV lost only approximately 20% of its original infectiousness, while all other types of influenza viruses were completely inactivated. Finally, replacement of the hemagglutinin (HA) and neuraminidase (NA) proteins of a temperature- and acid-sensitive IAV with the hemagglutinin-esterase fusion (HEF) protein of a stable IDV through a reverse genetic system largely rendered the recombinant IAVs resistant to high-temperature and low-pH treatments. Together, these results indicated that the HEF glycoprotein is a primary determinant of the exceptional temperature and acid tolerance of IDV. Further investigation into the viral entry and fusion mechanism mediated by the intrinsically stable HEF protein of IDV may offer novel insights into how the fusion machinery of influenza viruses evolve to achieve acid and thermal stability, which as a result promotes the potential to transmit across mammal species.

**IMPORTANCE** Influenza D virus (IDV) utilizes cattle as a primary reservoir. Increased outbreaks in pigs and serological evidence of human infection have raised a concern about the potential of IDV adapting to humans. Here, we directly compared IDV’s stability to that of other influenza types (A, B, and C) following prolonged incubation at high temperatures or in a low-pH environment. We found that IDV is the most stable of the four types of influenza viruses. Importantly, we demonstrated that the hemagglutinin-esterase fusion (HEF) protein, which drives the fusion between viral and host cell membranes, is the primary determinant for the high thermal and acid stability of IDV. Considering that there is a link between the acid stability of the hemagglutinin protein of influenza A virus and its cross-species transmission, further investigation of the mechanism of HEF-directed viral tolerance may offer novel insights into tissue tropism and cross-species transmission of influenza viruses.

## Abstract (precis)

Influenza D virus (IDV) utilizes cattle as a primary reservoir. Increased outbreaks in pigs and serological evidence of human infection have raised a concern about the potential of IDV adapting to humans. Here, we directly compared IDV’s stability to that of other influenza types (A, B, and C) following prolonged incubation at high temperatures or in a low-pH environment. We found that IDV is the most stable of the four types of influenza viruses. Importantly, we demonstrated that the hemagglutinin-esterase fusion (HEF) protein, which drives the fusion between viral and host cell membranes, is the primary determinant for the high thermal and acid stability of IDV. Considering that there is a link between the acid stability of the hemagglutinin protein of influenza A virus and its cross-species transmission, further investigation of the mechanism of HEF-directed viral tolerance may offer novel insights into tissue tropism and cross-species transmission of influenza viruses.

## OBSERVATION

Influenza viruses are segmented, single-stranded, negative-sense, enveloped RNA viruses and belong to the *Orthomyxoviridae* family. Four types of influenza viruses, designated influenza A virus (IAV), influenza B virus (IBV), influenza C virus (ICV), and influenza D virus (IDV), have been identified. The genomes of IAV and IBV consist of eight RNA segments, whereas ICV and IDV have only seven segments. IAV and IBV contain two major surface glycoproteins: hemagglutinin (HA), which binds to sialylated host cell receptors and mediates membrane fusion, and neuraminidase (NA), which prevents the HA from host cell membrane engagement by cleaving sialic acids from receptors, thereby releasing newly assembled virus particles (1). ICV and IDV, however, have only one major surface glycoprotein, the hemagglutinin-esterase fusion (HEF) protein, which performs all above functions, including receptor binding, receptor destroying, and membrane fusion (2). IAV infects avian, human, swine, and many other mammalian species, including tigers, seals, dogs, and horses, while IBV and ICV are found principally in humans and rarely infect other species. The recently discovered IDV causes respiratory diseases primarily in cattle and to a lesser extent in pigs. Moreover, serological evidence for IDV infection in small ruminants and humans has been established (3, 4). Since the initial isolation of IDV in the United States in 2011, IDV has been reported in China, Mexico, France, Italy, and Japan. Under experimental conditions, IDV is capable of infecting ferrets and guinea pigs and transmitting to naive animals by direct contact (2, 5).

IDV differs from all historically known influenza viruses in that it utilizes cattle as a primary reservoir. Adaptation to cattle may confer some unique features to IDV, which enhance its survival in this particular agricultural animal population. Such inherited characteristics through evolution may make IDV distinguishable from other types of influenza viruses circulating in ducks, pigs, or humans. As a first step toward identifying novel biological traits and better understanding the infection biology of newly emerging IDV, we directly compared the stability and infectivity of IDV to those of other influenza types following exposure to either high temperatures or low-pH solution. Interestingly, our experiments revealed that IDV was more resistant to high temperatures and highly acidic environments than the other three types of influenza viruses. Significantly, we found that the viral HEF glycoprotein is a primary force in dictating the exceptional stability of IDV infectivity at low pH and high heat.

**FIG 1:**
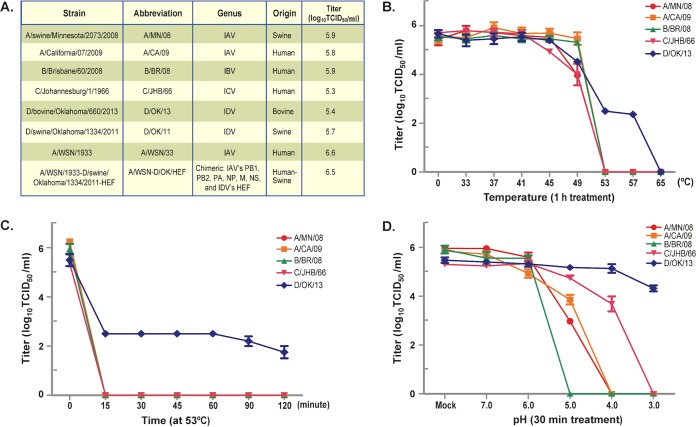
Thermal and pH stability of influenza viruses. (A) A list of the influenza viruses used in this study and their infectivity determined as TCID_50_ per ml in MDCK (Madin-Darby canine kidney) cells. These viruses were replicated in MDCK cells under the same conditions. (B) All viruses were treated in solution under different temperatures for 60 min and incubated for another 30 min in 4°C prior to the infectivity experiment. Note that the virus titers were 5.4 log_10_ TCID_50_/ml for A/MN/08, 5.6 log_10_ TCID_50_/ml for A/CA/09, and 5.6 log_10_ TCID_50_/ml for B/BR/08, which are slightly different from those used for the experiments shown in panels C and D. (C) A/MN/08, A/CA/09, B/BR/08, C/JHB/66, and D/OK/13 were treated in solution under 53°C for different time points and incubated for another 30 min in 4°C prior to the infectivity experiment. (D) A/MN/08, A/CA/09, B/BR/08, C/JHB/66, and D/OK/13 were treated in solution with different pH values for 30 min at room temperature, followed by neutralization and incubation for another 30 min in 4°C prior to the infectivity experiment. The data presented in this figure are representative of three independent experiments, with each assay sample tested in duplicate. The error bars represent standard deviations and indicate the variations among the experiments.

The viruses used in this study are listed in Fig. 1A, including two IAVs (originated from swine and human), one IBV (from human), one ICV (from human), and two IDVs (from cattle and swine). We started by investigating the effect of temperature on the stability and infectivity of the influenza viruses. Temperature gradients evaluated included 33°C, 37°C, 41°C, 45°C, 49°C, 53°C, 57°C, and 65°C. A temperature of 0°C was included as a control. All the viruses listed in Fig. 1A were treated under this set of temperatures for 1 h at neutral pH and then incubated on ice for another 30 min. After treatment, the infectivity of these viruses was determined by measuring viral 50% tissue culture infective doses (TCID_50_s) in MDCK (Madin-Darby canine kidney) cells using the standard protocol (6, 7). Viral titers for all tested viruses were not impaired when treated at 33 to 41°C but started to decline at 49°C (Fig. 1B). The 53°C treatment clearly discriminated IDV D/OK/13 (see Fig. 1A for the influenza virus strains and influenza virus abbreviations) from the three other types in that IDV retained a high residual infectivity (~2.5 log units of TCID_50_/ml), while IAV, IBV, and ICV were completely inactivated. Remarkably, IDV was still infectious when treated at 57°C, and the complete loss of its infectivity was observed only after 1 h of incubation at 65°C.

We next selected the 53°C treatment condition at neutral pH with different time points after incubation (0, 15, 30, 45, 60, and 120 min) to directly study the thermal stability and infectivity of all types of influenza viruses (Fig. 1C). Significantly, only D/OK/13 was able to tolerate the high temperature of 53°C. All other types of influenza viruses, such as A/MN/08, A/CA/09 (pdm09HIN1), B/BR/08, and C/JHB/66, completely lost their capacity to survive when exposed to 53°C for 15 min (Fig. 1C). Notably, D/OK/13 was able to maintain about 40% of its infectious titer for 2 h (Fig. 1C). These results suggested that IDV is the most temperature-stable influenza type of all influenza virus types.

In parallel, we directly studied the stability and infectivity of these viruses (Fig. 1A) at different pH values ranging from 3.0 to 7.0. For pH trials, 0.2 M sodium acetate-acetic acid buffer was adjusted from pH 3.0 to 7.0 at 1-unit increments with the addition of 10% HCl. A nontreated control (NTC) was also included in the analysis. All pH trials were completed at room temperature for 30 min. Each pH treatment was measured at the start of the study and confirmed at the completion of each trial. In all cases, it did not vary more than 0.2 unit from the starting pH value. Then, treated viruses were pH neutralized with infection medium and incubated for additional 30 min at 4°C followed by measuring their TCID_50_ in MDCK cells using the same protocol described above. Remarkably, D/OK/13 was determined to be the most stable influenza virus when treated with a pH of as low as 3.0 (Fig. 1D). The virus retained about 80% of its original infectivity at pH 3.0, whereas all other types of influenza viruses completely lost their infectivity at a pH of 3.0 (Fig. 1D). Interestingly, IBV was found to be the most unstable influenza virus and lost its infectivity at pH values below 5.0 (Fig. 1D), followed by the two IAVs (completely inactivated at pH 4.0). It is interesting to note that ICV, evolutionarily and genetically close to IDV, also acquired appreciable resistance to more-acidic environments. For example, despite becoming noninfectious at pH 3.0, ICV retained approximately 60% of its infectivity at pH 4.0. Overall, the ranking in order of the inherent stability at low pH is as follows: IDV > ICV > IAV > IBV. Together, the results of our experiments indicated that IDV is the most stable influenza virus in a low-pH environment.

**FIG 2:**
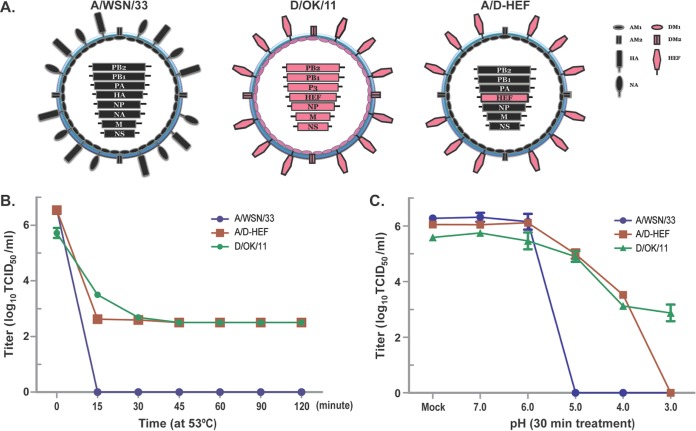
HEF is a key determinant of the exceptional acid and temperature stability of IDV. (A) Schematic representation of A/WSN/33, D/OK/11, and chimeric A/D-HEF viruses used in this study. Specifically, we generated a D/OK/11 HEF expression plasmid in the context of pHW2000-derived dual-promoter reverse genetic system (RGS) expression construct of the A/WSN/33 neuraminidase (NA) segment. The complete HEF cDNA from D/OK/11 is flanked by 183 nucleotides of the 3′ NA viral RNA (vRNA) and 157 nucleotides of the 5′ NA vRNA, and initiation codons in the 3′ NA vRNA are mutated to express HEF protein only. Chimeric A/D-HEF virus was generated through cotransfection of 293T and MDCK cells with the chimeric HEF plasmid together with A/WSN/33-derived PA, PB1, PB2, NP (nucleoprotein), M (matrix), and NS (nonstructural) RGS plasmids. (B) A/WSN/33, IDV D/OK/11, and chimeric A/D-HEF were treated in solution at 53°C for different times, followed by incubation in 4°C for 30 min prior to infection experiments. (C) A/WSN/33, D/OK/11, and chimeric A/D-HEF were treated in solution over a range of pH values from pH 3.0 to 7.0 for 30 min, followed by neutralization and incubation for another 30 min in 4°C prior to the infectivity experiment. The data presented in this figure are representative of three independent experiments, with each assay sample tested in duplicate. The error bars represent standard deviations and indicate the variations among experiments.

Premature activation of viral fusion peptide is detrimental to influenza A virus infectivity (8). Previous studies have established that pretreating IAV prior to infection of cells by exposing the virus to high temperature or low pH can cause premature exposure of the viral fusion peptide in the HA protein, which leads to an irreversible loss of viral infectivity (8, 9). Interestingly, IDV and to a lesser extent ICV, exhibiting a good acid stability (Fig. 1D), both harbor the HEF protein on the virion surface. The HEF protein in IDV and ICV, like HA in IAV and IBV, mediates virus entry and virus-cell membrane fusion in intracellular endosome compartments in an acidic environment. On the basis of the above analysis, we hypothesize that the viral HEF glycoprotein is a primary determinant of the thermal and acid stability of IDV. To test this hypothesis, the HA and NA segments of an IAV H1N1 WSN/1933 (A/WSN/33) were replaced with the HEF segment of D/OK/11 (from swine) using the reverse genetic system (Fig. 2A), and the stability and infectivity of the rescued A/D-HEF chimeric virus were examined and directly compared with its parental viruses (A/WSN/33 and D/OK/11) by the protocol discussed above. As demonstrated in Fig. 2B, A/WSN/33 (wild type) completely lost its infectivity when treated at 53°C for 15 min. In marked contrast, the A/D-HEF chimera was able to survive and maintain its infectious titer after exposure to 53°C for up to 120 min, which was the same as for wild-type D/OK/11 (the HEF protein donor). Furthermore, following exposure to different pH environments from pH 3.0 to 7.0, the A/WSN/33, like other two IAVs (Fig. 1D), completely lost its infectivity after exposure to pH 5.0 or below (Fig. 2C). Remarkably, at pH 5.0, the A/D-HEF chimera lost only about 15% of its infectivity. The chimera still retained more than 50% of its infectivity at pH 4.0 but was completely inactivated only at pH 3.0. Similar to D/OK/13, which originated in cattle (Fig. 1D), D/OK/11 (swine origin) also survived when held in solution for 30 min at pH 3.0. In summary, these results suggested that the HEF protein is a key factor in determining IDV’s exceptional plasticity in response to high-temperature stress and a low-pH environment. It should be noted that the HEF protein, in the context of the A/D-HEF chimeric virus, did not make the chimera fully gain the acid resistance trait as demonstrated in wild-type D/OK/11 (Fig. 2C) or D/OK/13 (Fig. 1D). This discrepancy may be caused by the subtle difference of the density and spatial arrangement of the HEF protein on the virion surface between the chimeric IAV and native IDV, which will be investigated in future studies.

Intriguingly, acquisition of this remarkable physicochemical stability has apparently not inhibited the ability of IDV to spread efficiently in global animal herds. Such stability may give IDV additional advantage in surviving well in harsh environmental conditions, such as heat and low pH. Relating both thermal and acidic stability of IDV to the HEF protein is very similar to recent results with the HA protein of IAV where it was shown that some HA mutations lowering the pH threshold in activation of the HA fusion peptide (i.e., increased acid resistance) evidently rendered the IAVs more resistant to heat (10–14). As such, the HA protein of IAV appears to follow IDV in gradually acquiring acidic and thermal stability. Although the mechanism is still largely unknown, it is generally believed that some animal IAVs (swine or poultry) conducting a virus-cell membrane fusion event at a lower pH can transmit more efficiently to humans than those fusing at a higher pH (15). Therefore, the increased acid stability and thermostability of HA have been viewed as important requirement, in addition to the receptor binding specificity/affinity, for efficient airborne transmission of IAVs from animals to humans. A recent work also showed that the neuraminidase protein of the 1918 pandemic IAV is relatively stable at low pH, and this low-pH stability is implicated in enhancement of virus replication (16). In light of the above facts, further investigation of the molecular mechanism by which naturally stable IDV enters the cell and fuses with the host membrane may offer novel insights into how the fusion machinery of influenza viruses in general evolves, directed by viral glycoproteins, to achieve higher acid and thermal stability, which as a result promotes the cross-species transmission potential between mammals.

The observation of viral HEF protein conferring exceptional resistance to high temperature and low pH raises several interesting questions with respect to the entry pathway and biology of IDV. First, can acidification artificially transform IDV into a fusogenic stage (i.e., fusion peptide completely exposed)? If so, we would anticipate that low-pH-induced fusogenic IDV would not be infectious. On the basis of our data that the pretreatment of IDV in a low-pH buffer prior to incubation with cells had no substantial effect on viral infectivity, while this treatment completely inactivated IAV, we speculate that low pH is required but not sufficient to trigger complete activation of the IDV fusion peptide, although we cannot rule out the possibility that activation of IDV’s fusion activity is pH independent or that IDV HEF conformational changes triggered by low pH leading to viral fusion are reversible (instead of IAV-like irreversible). Second, is there a requirement for cellular acidification in IDV entry? Previous studies have demonstrated that IDV-related ICV requires a low-pH-dependent fusion in cells (17, 18). Interestingly, an earlier ICV fusion characterization study revealed that ICV fused with *in vitro*-reconstituted liposomes relatively slower than IAV or IBV did (17). The time delay in fusion may reflect the more stable nature of the HEF protein of ICV in a low-pH environment, which is demonstrated in our study (Fig. 1D). On the basis of the results of the above analysis, we speculate that IDV will likely utilize a low-pH-dependent endocytosis route, the common pathway for all influenza viruses, to enter the cell and fuse with the endosomal membrane to initiate infection. Third, what is the primary mechanism employed by IDV to activate the virus-cell membrane fusion event? Here, we propose three models for IDV fusion mechanism. The first test model is that in addition to low pH, receptor binding may play a critical role in priming IDV fusion, a mechanism used by avian sarcoma and leucosis virus (ASLV) (19), Jaagsiekte sheep retrovirus (20), and hepatitis C virus (21). It is interesting to note that ASLV, like IDV, is resistant to inactivation by low pH. The second model is that some cellular factors in acidic intracellular compartments may be required for activation of IDV fusion as observed for Ebola virus and severe acute respiratory syndrome coronavirus (SARS-CoV) (22, 23). The third model is that intracellular processing of IDV could trigger some changes in the trimeric structure of the HEF protein, which in turn activates the IDV fusion peptide and drives the virus-cell membrane fusion. This model has been previously suggested for acid-resistant bovine pestivirus in activation of pH-triggered fusion during viral entry (24). Further investigation of these hypothetical models is needed to achieve a better understanding of the entry and fusion mechanisms mediated by the intrinsically stable HEF protein of IDV. Such information may offer novel insights into how the fusion machinery of influenza viruses in general evolves to achieve the acid and thermal tolerance, which as a result promotes the potential to transmit across mammal species.
